# Tweeting about twenty: an analysis of interest, public sentiments and opinion about 20mph speed restrictions in two UK cities

**DOI:** 10.1186/s12889-021-12084-x

**Published:** 2021-11-05

**Authors:** Tushar Semwal, Karen Milton, Ruth Jepson, Michael P. Kelly

**Affiliations:** 1 School of Engineering, University of Edinburgh, Edinburgh, UK; 2 Norwich Medical School, University of East Anglia, Norwich, Norfolk UK; 3 Scottish Collaboration for Public Health Research and Policy, University of Edinburgh, Edinburgh, UK; 4 Department of Public Health and Primary Care, University of Cambridge, Cambridge, UK

**Keywords:** Pubklic health, Policy, Intervention, Speed restrictions, Social media, Twitter mining, Sentiment analysis

## Abstract

**Background:**

Twenty miles per hour (20mph) speed limits (equivalent to roughly 30kmh) have become part of public health policies to reduce urban road collisions and casualties, especially in Western countries. Public opinion plays a crucial role in opposition to and acceptance of policies that are advocated for improving public health. Twenty miles per hour speed limit policies were implemented in Edinburgh and Belfast from 2016 to 2018. In this paper, we extract public opinion and sentiments expressed about the new 20mph speed limits in those cities using publicly available Twitter data.

**Methods:**

We analysed public sentiments from Twitter data and classified the public comments in plain English into the categories ‘positive’, ‘neutral’, and ‘negative’. We also explored the frequency and sources of the tweets.

**Results:**

The total volume of tweets was higher for Edinburgh than for Belfast, but the volume of tweets followed a similar pattern, peaking around 2016, which is when the schemes were implemented. Overall, the tone of the tweets was positive or neutral towards the implementation of the speed limit policies. This finding was surprising as there is a perception among policymakers that there would have been public backlash against these sorts of policy changes. The commonly used hashtags focused largely on road safety and other potential benefits, for example to air pollution.

**Conclusions:**

Overall, public attitudes towards the policies were positive, thus policymakers should be less anxious about potential public backlash when considering the scale-up of 20mph speed restrictions.

## Introduction

With more than one million individuals dying each year on the road [1], reducing road casualties is a public health priority. Twenty miles per hour (20mph) speed limit policies (equivalent to roughly 30kmh) have become a part of public health policies to reduce urban road collisions and casualties, especially in Western countries [2, 3]. Twenty mph limits are predominantly sign-based measures to reduce motor vehicle speed and are mainly used in residential areas. ‘Limits’ are distinct from ‘zones’ which use physical infrastructure such as speed bumps or chicanes. A 2020 review concluded that 20mph ‘zones’ are effective in reducing collisions and casualties; however, there was insufficient evidence to draw robust conclusions on the overall public health effectiveness of limits [4]. The cost of installing and maintaining physical infrastructure makes the scale-up of 20mph zones expensive and therefore politically challenging to justify. The relative cost of installing and maintaining signs and lines, plus some legislative, educational and enforcement activities, may make 20mph limits simpler and less expensive, and more feasible to implement at scale.

From 2016 to 2018, the city council in Edinburgh and the Devolved Administration in Belfast implemented new 20mph speed restrictions. The National Institute for Health Research (NIHR) funded an evaluation of the two schemes (grant number: 15/82/12; full details can be found in the final project report [5]). One element of the evaluation explored the political decision-making processes that led to the implementation of these schemes. This involved: investigating the official records of Edinburgh City Council and the Department for Infrastructure in Northern Ireland; conducting interviews with a range of key actors and stakeholders in both cities; and examining local press coverage. While these methods proved informative, they typically revealed a particular storyline, which is the narrative that council officials or others wanted to portray. The findings found little real reported antagonism or animosity towards the initiatives.

Public opinion plays a crucial role in opposition to and acceptance of policies like speed restrictions. However, limited research has sought to understand broad public attitudes towards 20mph speed limits. What is known about public attitudes largely comes from reviews of official records in the form of responses to public consultations, which is likely to reflect a narrow range of perceptions from the sub-sample that responded. This research sought to explore whether social media content could provide insight into wider public perceptions of 20mph speed limit interventions. While the research was exploratory in nature, our working hypothesis was that the introduction of 20mph policies would generate opposition, and that one of the fora in which this opposition would be expressed, would be social media.

Social media provides a platform for people to share publicly views and opinions on a wide range of issues. It may therefore provide a useful tool for gaining greater insight into the public’s reaction to the proposed schemes, and importantly if and how these reactions changed over time. An advantage of using social media data, over for example a questionnaire, is that social media typically reflects reactions to events in real-time. This is important, given the transient nature of perceptions, attitudes and emotions.

Twitter is a micro-blogging platform, which provides a useful window on aspects of current public sentiments. Twitter can be a rich source of information as its users openly and often candidly express their views and opinions on agendas or policies. This information can be anonymously “mined” and provide a valuable insight into public sentiment at any one time or duration. The extracted sentiments can then help to inform understandings of reactions to the government plans or implementation of policies. This kind of analysis could lead to better preparation for future implementation of similar policies in the UK.

The Belfast city centre scheme came into force in February 2016 and was implemented in a single phase [6]. Edinburgh implemented the city-wide 20mph speed limit network between July 2016 and March 2018. The scale up of 20mph limits was implemented in four phases across seven areas of Edinburgh, with each taking approximately 16 weeks to put in place. The aim of this paper is to explore public opinion about these 20mph schemes through mining Twitter data and undertaking sentiment analysis. Specifically, we were interested in identifying broader public reactions to the speed restrictions than might be captured via responses to formal public consultations. Our research question was: What was the public’s reaction to the Edinburgh and Belfast 20mph policies, as expressed through Twitter?

In this paper, we firstly explain the concept of sentiment analysis, before describing the methodology, including the collection of Twitter data and the steps involved in undertaking sentiment analysis of the data. We then present both statistical analysis and sentiment extraction. The results include the total number of tweets, tweets per year, and the most used hashtags, as well as the sentiment of the tweets in terms of being positive, negative or neutral. Finally, we discuss the findings in relation to the Edinburgh and Belfast 20mph policies and implications for future policies of this kind.

### Theoretical framework

#### Sentiment analysis

Sentiment analysis is a process of automatically extracting emotions, attitudes, views, and opinions from the text data, by using techniques from Natural Language Understanding (NLU). Sentiment analysis generally classifies text into categories such as positive, neutral, or negative. It is sometimes referred to as opinion mining or appraisal extraction. Though sentiment analysis provides an automated method to extract public opinions, it cannot replace traditional survey methods, but it can work in a complementary fashion [7].

There has been a plethora of papers on analysing public sentiments using Twitter data for various subjects including politics, environment, health, and the COVID-19 pandemic. Chen et al. [8] proposed a technique to classify student problems through the exchange of comments on Twitter. The authors implemented a multi-label classification algorithm to classify students’ problems through tweets. The authors reported that their work was the first to show how informal social media content can provide insights into students’ experiences. Bahrainian et al. [9] presents a hybrid method for polarity detection in the consumer-products domain. The proposed method leverages Sentiment Lexicon to generate a fresh set of features to train a linear machine learning classifier. The paper illustrates that the hybrid algorithm outperforms a unigram-based classification algorithm. Similar product review studies have been proposed, using batches of machine learning methods and semantic analysis [10]. The authors first collected online user reviews from tweets, pre-processed the dataset, and then extracted adjectives to form a feature vector. Different machine learning techniques based on probability and linear modelling were applied to the resulting feature vector to classify the reviews as positive or negative. Sehgal et al. [11] presented a method to automatically predict stock prices using web sentiments. The proposed system learns correlation between the stock values and the sentiments extracted from user messages on financial digital boards. A similar analysis is suggested for the e-learning domain [12] where an opinion mining method is developed to feedback from the candidates participating in such e-learning systems. The paper investigated three feature selection methods – mutual information (MI), information gain (IG), and computer-human interaction statistics, and demonstrated that IG exhibits the best performance for sentiment classification.

Both anomaly removal and classification of Twitter data have been studied [13] and several papers on analysis of public opinion on political parties and views have been described [14–16]. The previous work on political opinion mining involves analysing specific elections such as the 2010 US and 2012 Korean presidential elections. Techniques such as topic modelling, mention-direction based network analysis, and term co-occurrence retrieval were employed to analyse the contents. The studies clearly demonstrate that Twitter data is a valuable data resource to trace the changes in social issues. There are a few papers that focus on the study of event detection such as traffic information [17], hazards [18] and road accidents [19]. A study analysing public sentiments on urban transportation issues provides a similar motivation as to the work presented in the present paper [20]. The paper proposed an opinion mining method to analyse traffic-related tweets posted by the individual users. The publicly available location information from the tweets, along with the sentiment extracted, were used to evaluate the satisfaction of transportation users.

Sentiment analysis on Twitter data has also been applied in other areas, for example monitoring public feeling towards products and events in real-time [21]. That paper took a different approach and primarily focussed on different pre-processing methods which could increase the accuracy of a sentiment classification system. A total of six pre-processing methods were applied on five Twitter datasets. The experimental results found that pre-processing methods which expanded the acronyms and replaced the negation in tweets, performed best. Pre-processing steps, which included removing URLs, numbers or stop-words, did not have any effect on the performance of the sentiment classifier. In addition, Sentiment Analysis on Twitter has also been applied to extract restaurant reviews from the Yelp^1^ and TripAdvisor^2^ datasets [22, 23]. Sentiment analysis on social media may be used for novel applications such as analysing the effect of a celebrity’s endorsement of products [24], identifying human trafficking [25], and education [26].

As far as we are aware, Twitter data about public opinion have not been analysed in respect to 20mph speed limit policies in the United Kingdom. In this paper, we present a systematic study of publicly available tweets to extract public opinion and sentiments on the 20mph speed limit policies across Edinburgh and Belfast.

### Methodology

**Fig. 1:**
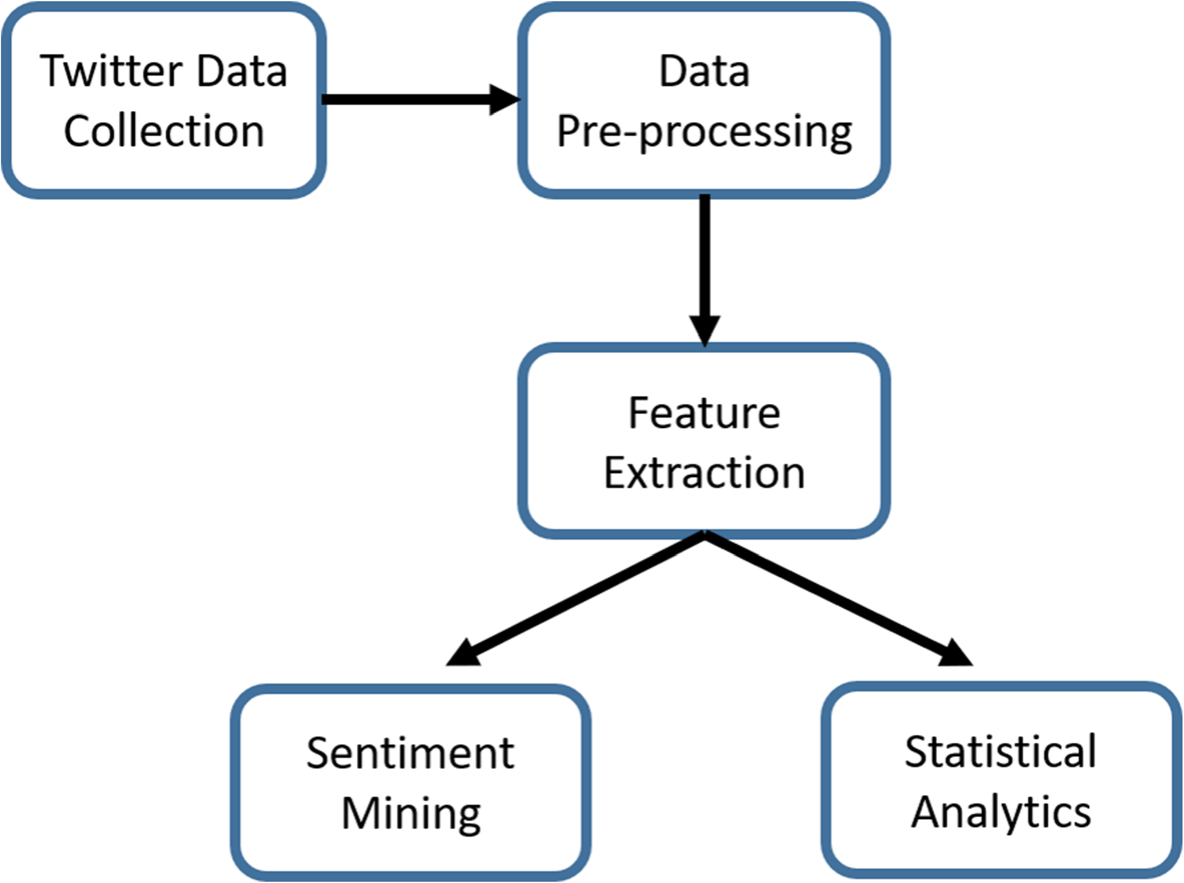
Pictorial representation of the methodology

Analysing public sentiments from Twitter data involves a series of procedures starting from data collection, then pre-processing, data mining, and interpretation. By geo-fencing our search to the regions of interest, we classified the public comments in plain English into the categories ‘positive’ (acceptance), ‘neutral’, and ‘negative’. We also explored the frequency and sources of the tweets. Figure 1 shows a block level architecture of our approach. We explain each of the steps below:

#### Data source and collection

We chose the micro-blogging service Twitter as the source of our data. With more than 15 million active users in the UK, Twitter is one of the most frequently used platforms for posting comments and messages. People can not only post new messages but also retweet already posted tweets, which makes it easy to support an idea behind the tweet. Twitter may also influence the public discussion about policy. The UK’s Prime Minister’s tweet on herd immunity during the Coronavirus pandemic was widely criticised by scientists and was not adopted as policy at the time.

Tweets for research purposes can be collected in three ways:

- Using freely available data repositories such as UCI,^3^ Kdnuggets,^4^ and SNAP.^5^
- Twitter Premium Application Programming Interface (API): Twitter provides multiple packages for APIs to collect tweets within a 30-day span or the full archive duration, where tweets starting from 2006 can be collected based on a set of query keywords. Apart from historical tweets, Twitter also provides stream APIs to collect tweets in real-time.
- More expensive options include tools such as Salesforce^6^ and Klear.^7^ These tools provide an automated solution to analyse the tweets.

The freely available repositories usually contain tweets about topics that are globally trending; for instance, the series of tweets on the COVID-19 pandemic and Black Lives Matter in 2020. There is much less activity concerning topics such as 20mph speed limit policies in Edinburgh and Belfast. Hence, we could not find any freely available data repositories containing tweets related to our topic of interest. Using automated services would have been another option, however they provide less flexibility for research and are more suitable for commercial purposes. In this paper, we have used the Full-Archive premium APIs to collect the tweets between January 2008 and September 2020. Twitter provides the premium APIs and the pricing is per the number of tweets streamed using the API.

Twitter premium APIs allows us to query tweets that contain desired keywords. For our work, we used keywords such as “20mph”, “speed limit”, “20 limit”, and joined them with the names of the cities – “Edinburgh”, and “Belfast”. This form of query provided the flexibility to search for any tweets that have a sensible variation of 20mph and the city name in any portion of a tweet. We collected a total of 24,000 raw tweets, across the two cities.

#### Pre-processing

Extracting information from the Twitter data is challenging. The data collected from Twitter APIs is raw with no filtering. The tweets have many idiosyncratic uses, such as emoticons, word repetitions etc. To categorise the tweets into sentiments, the data have to be pre-processed. The pre-processing task involves filtering URLs, stop words, removing hashtags (#) and other Twitter notations such as @, RT, and username. We performed the following steps to pre-process the data:

- Filter the URLS, emoticons, hyperlinks, and any non-alphabetical notations since we were focussed on the text comments.
- Remove the Twitter tags such as usernames (@), Retweet (RT), and hashtags (#).
- Filter stop-words such as ‘is’, ‘am’, ‘are’, etc. since they do not contribute to the sentiments in the text.
- Representations such as g8, f9, and happyyyy are slang, which emphasise emotions. We compressed and decompressed them such that g8, f9, and happyyyy are transformed to great, fine, and happy, respectively.

**Fig. 2:**
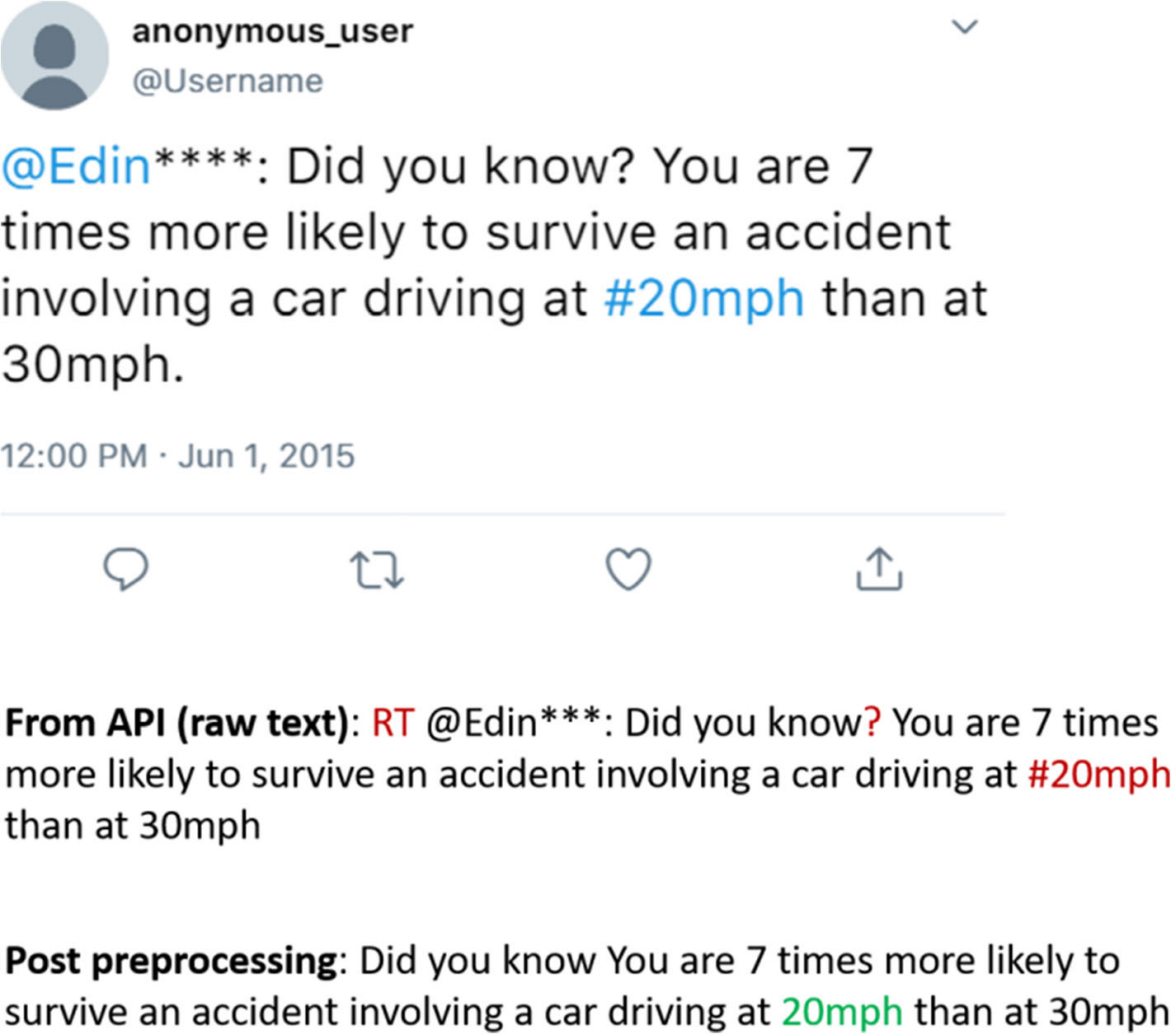
Shows a raw tweet and the same tweet after the pre-processing step

Figure 2 an anonymised sample tweet and the corresponding raw text after pre-processing. The blue text in the sample tweet is text that Twitter recognises as a handle or hashtag. In the processing steps, the red letters denote the part to be pre-processed while green is the filtered part

#### Data mining

We performed two forms of analysis on the pre-processed Twitter data. We first carried out the statistical analysis to look at patterns in the way the 20mph policies were viewed by the public since the discussions started on Twitter. We also mined for other statistical features such as number of tweets per year, most number of bi- and tri-grams used in tweets, and other forms of lexical analysis. In addition to the statistical analysis, our main goal was to understand public opinion through the exchange of tweets. We used Machine Learning (ML) techniques to train a model on a portion of full Twitter data collected and then used it to classify the tweets as positive, negative or neutral for the remaining Twitter data. We leveraged the pre-trained ML model, which had been already tuned to the sentiment classification problems. This form of ML technique is called Transfer Learning [27].

#### Dashboard and visualisation

**Fig. 3:**
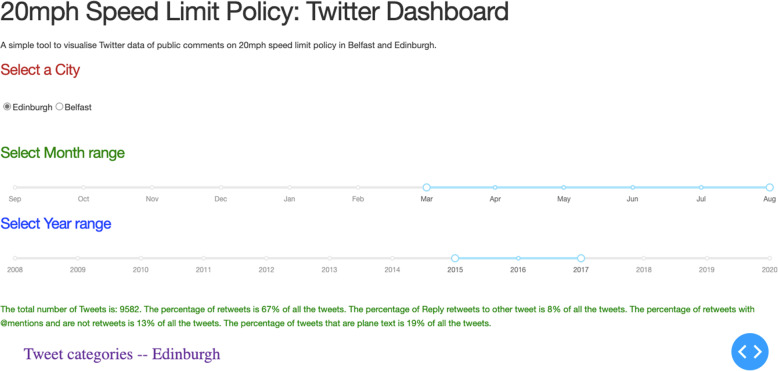
The dashboard which allows a user to set custom date ranges of comments that appeared on Twitter

We designed a Python and Flask based data visualisation dashboard using the Dash library. A snapshot of the dashboard is shown in Fig. 3. The dashboard provides an easy-to-use dynamic interface to filter the data as per the duration of time for which the user is interested. Given a range of dates, the dashboard presents several pieces of key information and statistics such as overall percentage of positive, negative, and neutral tweets and word-clouds, and displays a few examples of tweets from each category of emotions. The dashboard is easy to customise. The dashboard is the front end to the data stored in the MongoDB database, which we have used in this work. MongoDB provides an organised storage of our data such that it can be queried later for further analytics. This method provides an easy transfer of information for future projects.

## Results

In this section, we provide the results found from both the statistical and ML analysis. We found that discussions about implementing the speed limits started long before such times as they were due to be imposed, thus we collected the tweets occurring from 2008 until September 2020. This wide timeframe allows us to measure the growth of public opinion since Twitter began to be used widely among the general public. The positive sentiments suggest acceptance, while the negative comments represent discontent or opposition.

### Total tweets collected

**Fig. 4:**
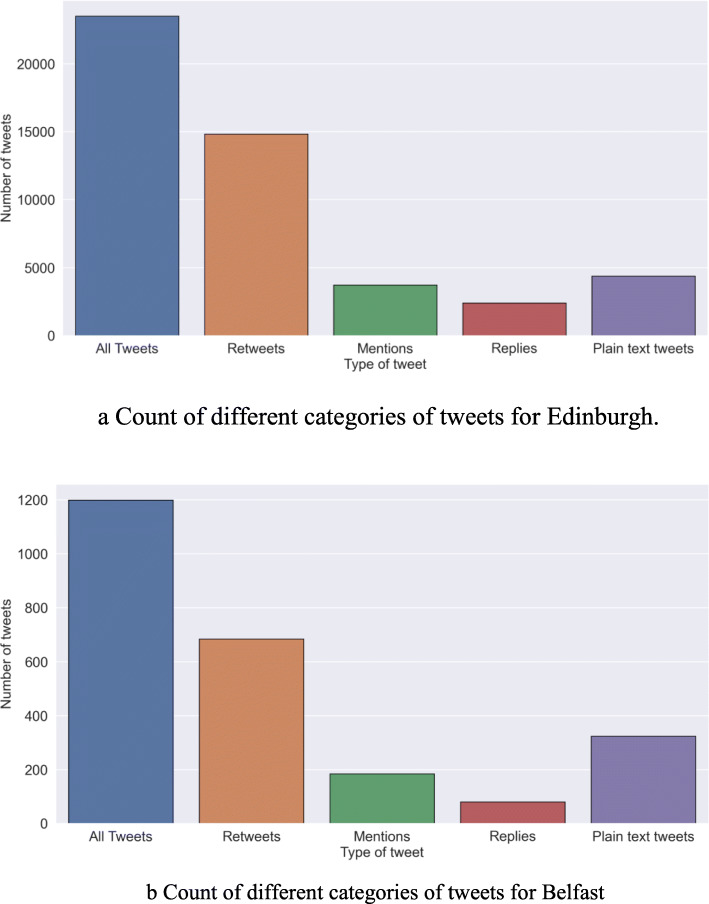
**a** Count of different categories of tweets for Edinburgh. **b** Count of different categories of tweets for Belfast

The total number of tweets indicates the volume of activity of people on the topic of 20mph on Twitter. Figure 4a and b show the total number of tweets collected from 2008 until September 2020 for Edinburgh and Belfast, respectively. As can be seen from the figures, the number of tweets collected for Edinburgh is much higher than that of Belfast, however, the proportion of Retweets, mentions, replies and plain text follows a similar pattern across both cities.

We found that the majority of tweets were the retweets of a few influential people who were the advocates for 20mph speed limit policies. A retweet denotes a positive vote for the message behind the tweets. These influential people span a range of professions including university researchers, reporters, scientists, and senior officials from advocacy organisations. Based on the number of retweets, it would seem strategic for policymakers to utilise influential people to sway public opinion on future transport policies.

### Tweets per year

**Fig. 5:**
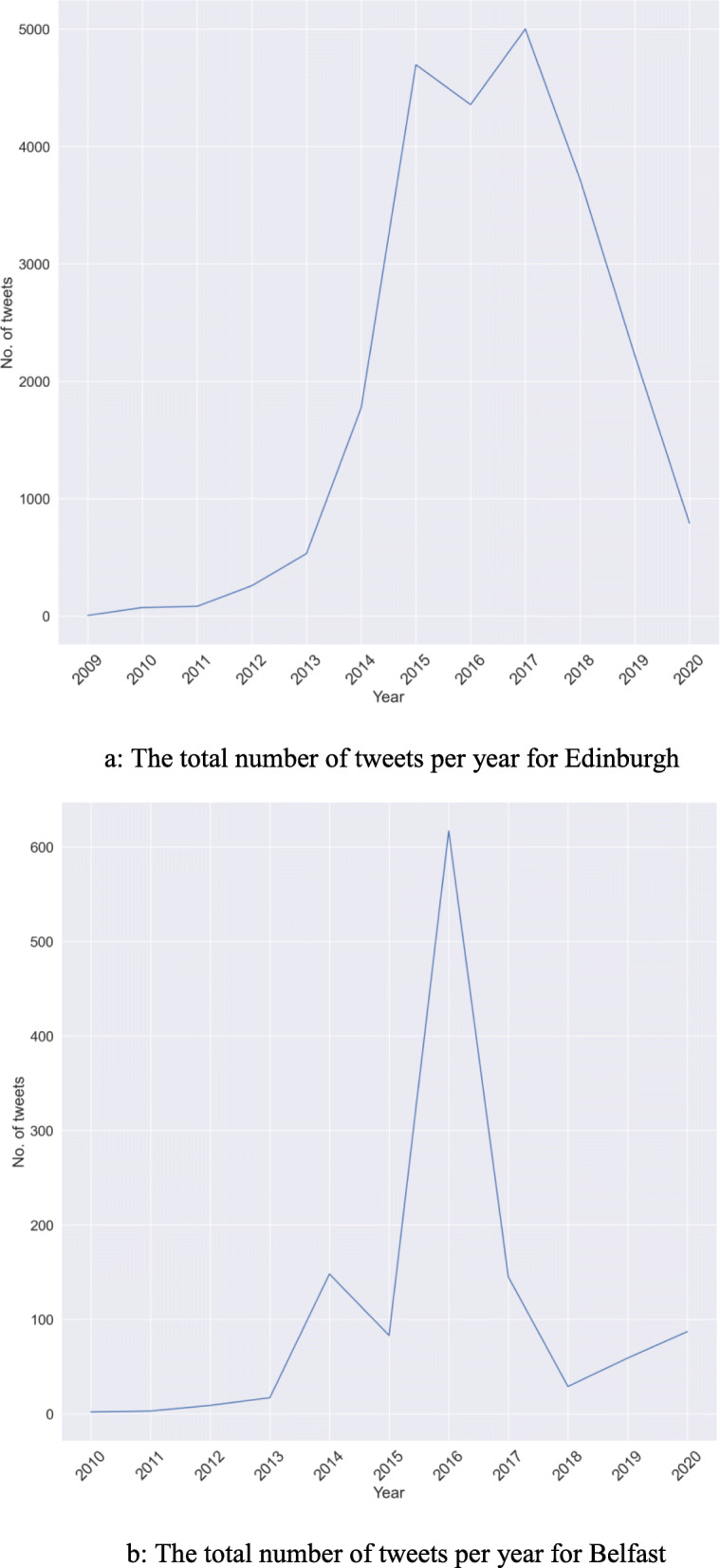
**a** The total number of tweets per year for Edinburgh. **b** The total number of tweets per year for Belfast

Since the implementation of the 20mph speed limit policies, there have been ongoing discussions about the topic on Twitter in both Edinburgh and Belfast. However, the plans for the intervention were laid several years before they were implemented. Hence, the count of Tweets both before and after policy implementation is an important factor that indicates awareness about the policies. Figure 5a and b show the count per year for Edinburgh and Belfast respectively. As can be seen, both figures show a gradual increase in the count of tweets on the topic from 2010 until it reaches a peak in 2016 for Belfast, the year of implementation, and 2017 for Edinburgh. The count decreases, apparently as people start adapting to the new policies. Analysing the counts before the peak and after the peak could be an interesting task since it informs on the acceptance and success of the policy. Specifically, analysing the negative emotions after the peak becomes more crucial than the positives, since it could help in extracting caveats, which may have gone unnoticed during the planning. Direct comments from the users is thus good feedback for any policy.

### Most used hashtags

**Fig. 6:**
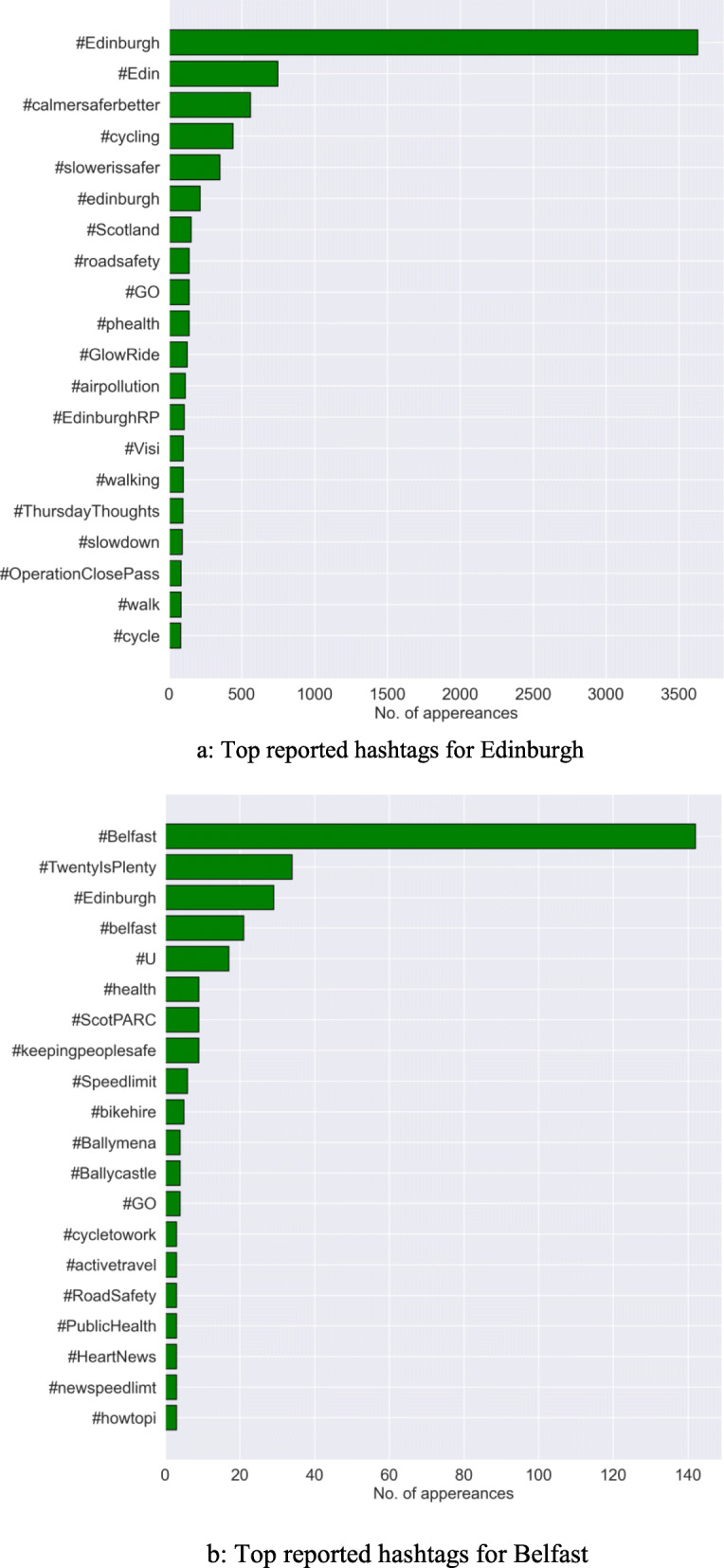
**a** Top reported hashtags for Edinburgh. **b** Top reported hashtags for Belfast

Hashtags are meta-data in a tweet representing a theme, topic, or a conversation context. For example, #BlackLivesMatter was a trending hashtag in the year 2016 and again in 2020. We extracted the hashtags and their count for each of the tweets and present a sorted representation in Fig. 6a and b for Edinburgh and Belfast respectively. These hashtags may represent the agenda behind each tweet on the 20mph topic in both cities. Thus, the information presents the core context on which the Twitter users are most focussed. The hashtag could denote a grievance, praise, or a general idea. Some of the most used hashtags for Edinburgh were found to be #calmersaferbetter, #cyling, #airpollution, and #roadsafety. Similarly, for Belfast, these were #keepingpeoplesafe, #activework, and #Twenty’sPlenty. As can be seen from Fig. 6a and b, none of the hashtags denote a negative sentiment, which points to the possibility that the general response to the policies among Twitter users was positive.

### Sentiment analysis

**Fig. 7:**
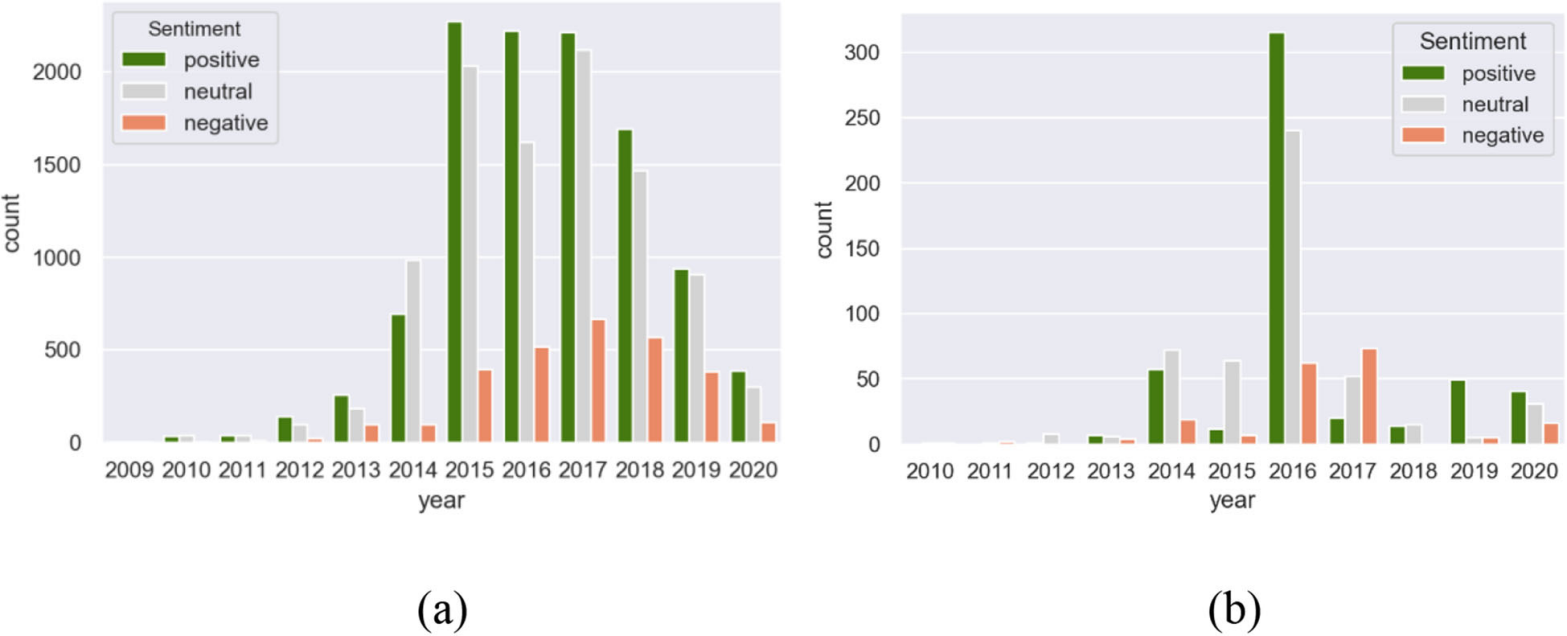
Variation in public sentiments per year for the cities of **a** Edinburgh and **b** Belfast

The foremost challenge in the Twitter data we collected is that they are unlabelled with respect to the sentiments. Since the data are not labelled, we leveraged the use of pre-trained models to classify our tweets into three categories – positive, neutral, and negative. We used a simple technique to assign sentiment scores to the tweets. The principle is to first tokenise the tweets into words and then assign a sentiment score to each of the words. The total emotion of the tweet is then the average of the emotions of the words. We verified the approach using two frequently used Python libraries – TextBlob^8^ and VADER^9^. To test the accuracy of the model used, we manually labelled 20% of the tweets and used them as the test dataset. Figure 7a and b show the sentiment graphs for tweets in Edinburgh and Belfast. As can be seen from the figures, the majority of the tweets are either positive or neutral. However, as the year 2016 approached the proportion of negative tweets increased with the other tweets. The next year to the one when the policy was implemented (2017), shows a further increase in negative sentiments. This year is significant since the public began to realise and see the impact of the 20mph speed limit policies on their daily lives.

**Fig. 8:**
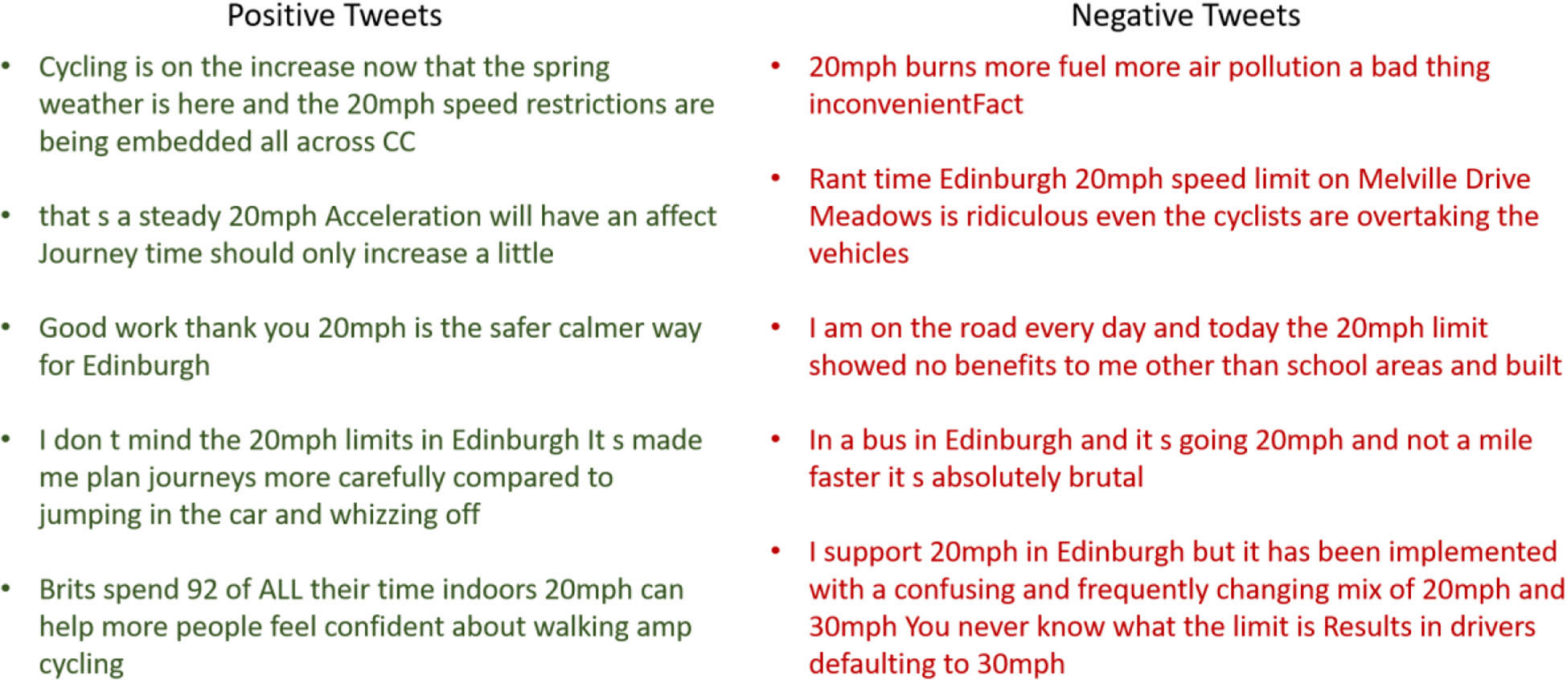
A few sample tweets classified into positive and negative categories

The number of tweets in Belfast was considerably fewer than that of Edinburgh, which reduces the effectiveness of the sentiment analysis. Lower Twitter usage related to the Belfast scheme may indicate lower awareness of the 20mph intervention among the public, or the public having less interest in the topic. A greater volume of tweets provides better insights into the sentiments of the public. Figure 8 provides a few tweets, each classified into either a positive or negative sentiment category. While some of tweets are quite clear, a few are ambiguous. For instance, the last tweet in the negative category is classified as negative, though the user supports the policy.

Similar to the analysis presented by Saura et al., in 2018 [28], we present the total number of positive, neutral and negative tweets across Edinburgh and Belfast in Table 1. These tweets span 2008 to 2020.

**Table 1:** Total number of positive, neutral, and negative tweets across Edinburgh and Belfast, which appeared on Twitter from 2008 to 2020

	Positive Tweets	Neutral Tweets	Negative Tweets
Edinburgh	10,874	9774	2858
Belfast	515	495	189

### Political agenda

Local authorities and Devolved Administrations are party political as well as bureaucratic organisations. We explored the extent to which tweets were party political and the degree to which the political parties engaged with Twitter on the policies. We collected the tweets from major political parties such as the Scottish National Party, the Liberal Democrats, and the Conservative Party in Edinburgh, and the Democratic Unionist Party and the Ulster Unionist Party in Belfast. We found that none of the political parties directly tweeted about the 20mph speed limits from their official Twitter accounts.

## Discussion

The aim of this paper was to collect and analyse the sentimental information in relation to Twitter activity about the 20mph speed limit interventions in Edinburgh and Belfast. The total volume of tweets was much higher for Edinburgh than for Belfast. This likely reflects a stronger focus on awareness raising and education in Edinburgh, where a public information campaign was an integral component of implementation. In contrast, public awareness raising efforts were relatively small scale in Belfast [29].

The volume of tweets followed a similar pattern, peaking around 2016, which is when the schemes were implemented, although the peak is much wider in Edinburgh, possibly reflecting the differences in process and implementation in the two cities. Edinburgh had delivered a pilot prior to the roll-out of the main scheme and scaled up the initiative over a much more extended period.

It is often assumed that social media is extremely powerful in affecting attitudes and opinion. This has led some public authorities to invest in working in social media, with Edinburgh Council being a good case in point. Our data does not suggest that this strategy was particularly successful in Edinburgh and there was very little engagement in Twitter activity in Belfast. Pressure groups and vested interest groups do use social media, as they did in the two cities, and in general the overall tone of the tweets was positive or neutral towards the implementation of the speed limit policies. This finding was surprising as there was a perception among policymakers that there was going to be public backlash against these transport policy changes. Twitter is a forum where one might expect these views to be openly expressed. In fact, most tweeters accepted the changes. The commonly used hashtags focused largely on road safety and other potential benefits, for example to air pollution.

We had anticipated at the outset that this analysis would give insight into the public’s opposition towards 20mph and would assist policymakers in better preparing for such negative responses in the future. This would put policymakers on the front foot in term of responding to opposition. What we found, however, was very little opposition among Twitter users. The findings clearly show that the majority of the public, or at least those who express views on Twitter, are supportive of 20mph and think these schemes should be implemented at scale. Concerns about the public’s reaction should not be viewed as a barrier to future adoption and implementation of such policies.

That said, the total volume of tweets in Belfast was relatively low and negative tweets exceeded positive tweets in Belfast in 2017. No such finding was observed for Edinburgh, where positive tweets far exceeded negative tweets at all time points. As mentioned, there was an integrated public awareness campaign in Edinburgh and the scheme there was rolled out area-by-area over many months. In contrast, limited public education efforts were implemented in Belfast and the scheme came into force over-night. It is quite possible that the introduction of 20mph came as a surprise to people living in Belfast, which resulted in public antipathy. The negativity was short-lived, and in fact zero negative tweets were identified in the following year.

This was a new area of investigation, which allowed us to explore public opinion on 20mph, as this is often not relayed in official reports. The methods used proved to be appropriate and could and should be utilized in other evaluations of policy decisions and public reactions. However, several limitations should be acknowledged. The primary drawback is that the data collected from social networking platforms such as Twitter are susceptible to noise, which affects the precision of analytic techniques such as the one used in our paper. Also, compared to questionnaires, using machine learning methods requires advanced training and knowledge of sophisticated tools. The current study focused on sentiment analysis. Other techniques, such as discourse analysis or the study of electronic word of mouth (eWOM), may provide additional - or even different - perspectives on the 20mph narrative, and would be useful to apply in future research on this topic [30, 31].

## Conclusions

In this paper, we analysed Twitter data on 20mph speed limit policies implemented in the cities of Edinburgh and Belfast. The study of social media data, especially for speed limit policies in the UK, is still in its infancy. The key aim of our work was to understand public opinion and sentiments about the effect of such policies in these cities. We presented both statistical and sentimental analysis of the data. The total volume of tweets was much higher for Edinburgh than for Belfast, although the volume of tweets followed a similar pattern. The commonly used hashtags focused largely on the benefits of 20mph for example on road safety and air pollution. Positive tweets far exceeded negative tweets; very little opposition among Twitter users was observed. The main implication is that policymakers should be less concerned about potential public backlash when considering the scale-up of 20mph speed restrictions. Implementing a public awareness campaign and rolling 20mph limits out progressively, may limit the potential for public push back in response to such policies. Finally, the methods used proved to be appropriate and have provided the first insight into public opinion in respect to 20mph speed limit policies in the United Kingdom, as expressed through Twitter. Similar approaches should be considered to advance understanding of the public’s attitude towards other public health interventions and policies.

## Data Availability

The data that support the findings of this study are available from Twitter. Restrictions apply to the availability of these data, which were used under license for this study. Data are available from the authors with the permission of Twitter.

## Footnotes

1 https://www.yelp.com/dataset

2 https://www.tripadvisor.com/

3 https://archive.ics.uci.edu/ml/index.php

4 https://www.kdnuggets.com/

5 https://snap.stanford.edu/data/

6 https://www.salesforce.com/

7 https://klear.com/

8 https://textblob.readthedocs.io/en/dev/

9 https://pypi.org/project/vaderSentiment/
