# Correction to: Tweeting about twenty: an analysis of interest, public sentiments and opinion about 20mph speed restrictions in two UK cities

**DOI:** 10.1186/s12889-021-12259-6

**Published:** 2021-11-26

**Authors:** Tushar Semwal, Karen Milton, Ruth Jepson, Michael P. Kelly

**Affiliations:** 1 School of Engineering, University of Edinburgh, Edinburgh, UK; 2 Norwich Medical School, University of East Anglia, Norwich, Norfolk, UK; 3 Scottish Collaboration for Public Health Research and Policy, University of Edinburgh, Edinburgh, UK; 4 Department of Public Health and Primary Care, University of Cambridge, Cambridge, UK

**Correction to: BMC Public Health 21, 2016 (2021)**

**https://doi.org/10.1186/s12889-021-12084-x**

It was highlighted that in the original article [1] the legend of Fig. 2 was erroneously swapped with an in-text sentence. This Correction article shows the correct Fig. 2. The original article has been updated.

**Fig. 2:**
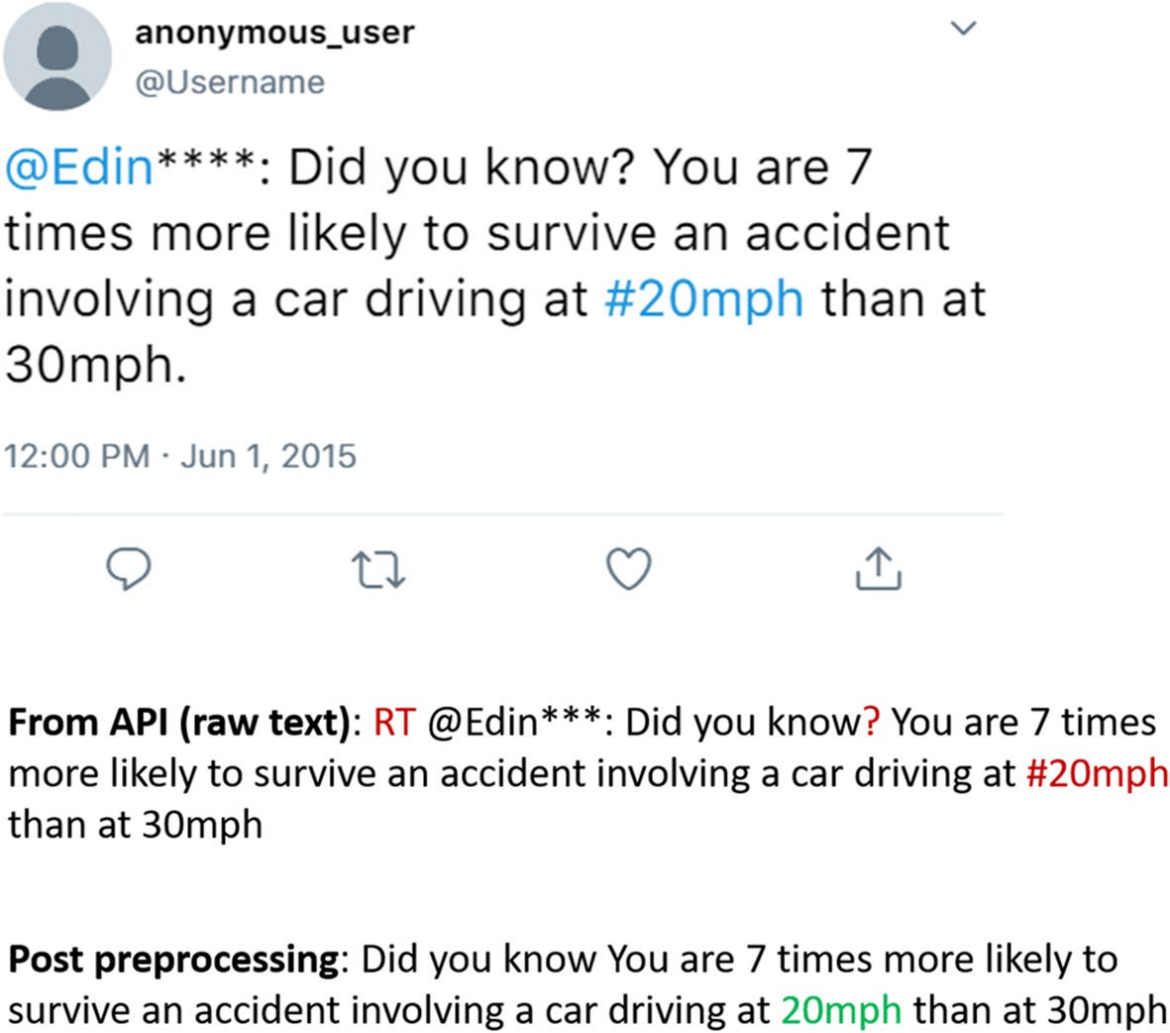
An anonymised sample tweet and the corresponding raw text after pre-processing. The blue text in the sample tweet is text that Twitter recognises as a handle or hashtag. In the processing steps, the red letters denote the part to be pre-processed while green is the filtered part
